# The Management of Acne in General Practice using Isotretinoin: A Scoping Review Protocol

**DOI:** 10.12688/hrbopenres.14215.1

**Published:** 2026-02-26

**Authors:** Shane Collins, Tony Foley, Diarmuid Quinlan, Emma Wallace, Laura Sahm, Aisling Farrell

**Affiliations:** 1 Department of General Practice, University College Cork School of Medicine, Cork, County Cork, Ireland; 2 Irish College of General Practitioners, Dublin, Ireland; 3 Pharmaceutical Care Research Group, School of Pharmacy, University College Cork School of Medicine, Cork, County Cork, Ireland

**Keywords:** Acne, Acne Vulgaris, Primary Care, General Practitioners, Isotretinoin

## Abstract

**Introduction:**

Acne Vulgaris (Acne) is a common, chronic, cutaneous disorder associated with scarring, hyperpigmentation, and adverse psychological effects. The high prevalence of acne places a significant burden on healthcare systems worldwide due to the cost of treatment. The high usage of antibiotics in the treatment of this condition also has a significant impact on the prevalence of antimicrobial resistance. Isotretinoin is considered to be the most effective treatment for the management of severe acne; however, issues remain with inequitable access to this drug. General Practitioners (GPs) are ideally placed to identify and manage patients with moderate-to-severe acne; however, in many countries the prescription of isotretinoin is limited to secondary care. To the authors' knowledge, no review has been conducted to identify, map and analyse literature pertaining to GPs’ use of isotretinoin for the management of acne.

**Methods:**

As recommended by the Joanna Briggs Institute (JBI), a three-step search strategy will be utilised for this scoping review. A search strategy will be developed using key words, search terms and controlled vocabulary suitable for each literature source. Both primary and grey literature will be eligible for inclusion in this review. Identified literature will be screened by two reviewers to identify relevant evidence. The full-text sources will then be evaluated by two independent researchers to extract relevant data. The findings of this review will be reported as a descriptive summary accompanied by a Preferred Reporting Items for Systematic Reviews and Meta-Analyses (PRISMA) flow diagram.

**Results:**

This scoping review protocol outlines a systematic approach to map a broad range of sources and summarise existing evidence regarding GPs’ use of isotretinoin for the management of acne in primary care.

**Conclusions:**

The objective of this scoping review is to comprehensively identify, map, and analyse literature pertaining to the management of acne using isotretinoin in general practice.

**Review registration:**

Registered on Open Science Framework on 02/10/2025 ( https://osf.io/rak3d/)

## Introduction

Acne Vulgaris (Acne) is a common inflammatory disorder of the pilosebaceous unit that affects a large proportion of young people, with up to 85% of 12-24 year olds experiencing some degree of acne. ^1, 2^ Although primarily seen in adolescence, it frequently persists into adulthood. ^2, 3^ Acne is associated with a significant impact on patients’ quality of life, contributing to increased self-consciousness and decreased psychological well-being, highlighting the broader personal burden of this disease. ^4^

Frequent and extended use of antibiotics in acne management has contributed to rising concerns over antimicrobial resistance, prompting current international guidelines to restrict antibiotic duration and encourage effective non-antibiotic therapeutic alternatives. ^5– 7^

Management of acne is also resource-intensive within healthcare systems. Many patients require repeated consultations, prolonged treatment courses and ongoing follow-up, contributing to workload in primary care, where most acne management occurs.

Clinical severity of acne varies, and approximately 15-20% of adolescents experience severe acne. ^8, 9^ Early and effective treatment is essential to reduce long-term complications, particularly permanent scarring, which can have lasting psychological and cosmetic consequences. ^10– 12^ Oral isotretinoin is widely regarded as the most effective treatment for severe and antibiotic resistant acne. ^13, 14^ However, isotretinoin prescription requires careful patient selection, counselling and monitoring due to its adverse effect profile, including the need to screen for mental health concerns and to monitor for teratogenic risks and blood dyscrasias throughout treatment. ^15, 16^

Internationally, there is considerable variation regarding the prescribing of isotretinoin. In some countries, prescribing remains dermatologist-led, most notably in the UK and USA. In contrast, several healthcare systems, such as Canada, Netherlands, Norway and New Zealand, support General Practitioner (GP) led prescribing. ^17– 21^ These differences reflect broader variation in national clinical pathways and distribution of prescribing responsibilities.

In Ireland, there are no current guidelines on prescribing isotretinoin in general practice. Moreover, no formal shared care protocols with specialist dermatology services or quality-improvement processes exist for GPs who prescribe isotretinoin. Prescribing is therefore largely dependent on individual GPs with an interest in dermatology opting to prescribe. ^17^ This lack of structured approach between primary and secondary care may lead to variation in clinical practice, potential gaps in safety monitoring and in the equity of access, particularly for females of childbearing potential.

Given their expertise in contraceptive management, mental health support and ability to provide continuity of care, GPs are well-positioned to prescribe and monitor isotretinoin. ^17^

Despite this, limited literature exists that explores the GP’s use of isotretinoin for acne in primary care. ^17, 22^

Emerging evidence suggests that while a proportion of GPs are willing to initiate isotretinoin, prescribing practices vary widely. Studies report that GPs often prescribe isotretinoin due to long dermatology waiting lists or personal interest in dermatology. ^17, 19^ However, significant gaps in knowledge and confidence remain with many GPs unfamiliar with guidelines, uncertain about safe monitoring or concerned about medicolegal and teratogenic risks. ^17, 19^ These studies also highlight suboptimal monitoring practices and inconsistent approaches to topical and antibiotic management. ^17, 23^ Nonetheless, a substantial proportion of GPs express interest in prescribing isotretinoin if supported by clearer guidelines and specialist input. ^17^

No review to date has provided a comprehensive synthesis of this disparate evidence to clarify the extent of these attitudes, barriers, behaviours, and practice variations across primary care settings.

A search of Google Scholar, JBI Evidence Synthesis, The Cochrane Database of Systematic Reviews, The Campbell Collaboration, PubMed and PROSPERO identified no existing or ongoing systematic or scoping reviews on this topic. This scoping review will therefore collate and map the available evidence on GP attitudes, practices, and challenges in prescribing isotretinoin in primary care.

### Review questions

What is known about GP’s roles, experiences, and practices in using isotretinoin for acne in primary care?

This scoping review aims to complete the following review objectives:

1. To map the existing literature describing how GPs prescribe, monitor, and manage isotretinoin in primary care.
2. To examine GPs’ views, knowledge, and perceptions regarding isotretinoin use for acne.
3. To identify challenges, safety concerns, training needs, and systemic factors affecting GP prescribing.

### Inclusion criteria

Population:

This review will consider all studies exploring GPs’ use of isotretinoin for the management of acne. GPs include primary care doctors and family medicine physicians.

Concept:

This scoping review will examine both peer reviewed and grey literature exploring the GP’s use of isotretinoin for the management of acne. Studies focusing on other acne treatments, or isotretinoin prescribed exclusively in secondary care, or for other conditions, will be excluded.

Context:

This review will consider literature examining isotretinoin use for acne for all patients in the primary care setting. Studies examining interventions in secondary care settings or interventions undertaken by physicians other than general practitioners will be excluded.

Types of sources:

This scoping review will consider peer reviewed qualitative, quantitative, and mixed-methods studies as well as grey literature. Studies published in any language will be included in this review.

Inclusion/Exclusion criteria summary:

A summary of the inclusion/exclusion criteria for this scoping review is available in Table 1 below.

**Table 1.** Inclusion/Exclusion criteria.

	Inclusion	Exclusion
**Population**	• Literature examining patients with acne in primary care settings	• Literature examining patients with acne in primary care by physicians other than GPs
**Intervention**	• Peer reviewed and grey literature examining isotretinoin for the management of acne	• Literature examining isotretinoin for the treatment of conditions other than acne • Literature examining interventions other than isotretinoin for the treatment of acne
**Study Design**	• All types of study design	• Incomplete trials (No results) • Duplicate publications of the same study that do not report on different outcomes
**Study Characteristics**	• All geographical regions and languages • No date restrictions	• None

## Methods

### Search strategy

As recommended by the JBI Manual for Evidence Synthesis 2024, a three-step search strategy was developed by the authors to locate all relevant literature that meets the threshold for inclusion in this review. ^24^ As part of this search strategy, an initial limited search using the MEDLINE (PubMed) database was performed. (01/10/2025) This initial search strategy, and all keywords used in this initial search, are included in Table 2 of this protocol. This initial search will be followed by a full search using identified key words and index terms in the subsequent comprehensive scoping review. This search strategy will be adapted to include index terms for each database included in the review. Finally, the reference lists of all included sources will be examined to identify any additional relevant literature. ^24^

**Table 2.** Search strategy: PubMed. Search undertaken on 01/10/2025.

Search	Terms	Results
#1	"Acne Vulgaris"[MeSH Terms] OR "Acne Vulgaris"[Title/Abstract] OR "Acne"[Title/Abstract]	24,730
#2	"general p*"[Title/Abstract] OR "gp"[Title/Abstract] OR "primary care*"[Title/Abstract] OR "primary healthcare*"[Title/Abstract] OR "family p*"[Title/Abstract] OR "General Practitioners"[MeSH Terms] OR "General Practice"[MeSH Terms] OR "Family Practice"[MeSH Terms] OR "Primary Health Care"[MeSH Terms] OR "physicians, primary care"[MeSH Terms]	808,124
#3	#1 AND #2	852

Relevant grey literature will be identified for inclusion in the review with the use of Google Scholar and Web of Science. The first 100 results from each source will be collated for inclusion in the review.

Following the identification of all relevant keywords, search terms, and controlled vocabulary, the search strategy will be finalised with the assistance of a university librarian and applied uniformly across all literature sources included in this review. This search strategy, and any variations, will be documented and reported in full in the final scoping review.

No language or date restrictions will apply to studies being considered for inclusion in this review. Google Translate will be used to translate any articles written in languages other than English.

The databases to be searched include MEDLINE (Ovid), CINAHL Plus with Full Text, APA PsycINFO, Scopus, Cochrane Library, and EMBASE. These databases were selected for their comprehensive coverage of medical, nursing, psychological, and pharmacological literature, ensuring a broad retrieval of relevant studies.

### Study selection

Following the completion of our search strategy, all identified abstracts will be collated and uploaded into Rayyan and duplicate citations removed. ^25^ The title and abstract of each study will be screened by two independent reviewers (SC, AF) for assessment against the inclusion criteria of this review. Relevant citations will be retrieved in full, and their full text will be assessed in detail against the inclusion criteria by the two independent reviewers. Reasons for the exclusion of sources of evidence at full text that do not meet inclusion criteria will be reported in the scoping review.

Any disagreements that arise between the reviewers at each stage of the selection process will be discussed and if necessary, resolved by a third reviewer (TF). The results of the search and the study inclusion process will be reported in full in the final scoping review and presented in a PRISMA flow diagram. ^26^

### Data extraction

Data will be extracted from papers included in the scoping review using a data extraction tool developed by the reviewers. Key information such as author(s), year of publication, journal, and results, or findings relevant to the review questions will be extracted using this tool. A draft data extraction tool is provided in Appendix B.

It is likely that the data extraction tool will be modified in an iterative fashion during the course of the review. Any modifications to the data extraction tool will be detailed in the scoping review. Any disagreements that arise between the reviewers will be resolved by discussion. If necessary, authors of papers will be directly contacted to request missing or additional data.

### Data analysis and results

Data gathered by this scoping review will be presented in table and figure format. Data such as the number of publications, study designs, year of publication, and country of origin will be analysed and presented. A descriptive summary which describes how the results relate to the review objectives and questions will accompany the results. As data analysis and presentation is an iterative process additional information that is relevant to the review objectives may be identified and included in the review, any deviations from the protocol such as this will be clearly identified in the scoping review.

## Data Availability

Open Science Framework: The General Practitioner’s Perspective on Isotretinoin for the Management of Acne Vulgaris in Primary Care: A Scoping Review Protocol. https://doi.org/10.17605/OSF.IO/SZKTB. ^27^ This project contains the following extended data:
• Appendix B - Draft Data Extraction Tool.xlsx. (Blank English version of the draft data extraction tool) Data are available under the terms of the Creative Commons Attribution 4.0 International license (CC-BY 4.0).
