# Supplementary material for: The Management of Acne in General Practice using Isotretinoin: A Scoping Review Protocol
Source: HRB Open Res. 2026 Feb 26;9:21. [Version 1] doi: 10.12688/hrbopenres.14215.1 (PMC13241837; doi:10.12688/hrbopenres.14215.1)
Supplement: Peer review history

# Reviewer report: Reviewer response for version 1

**DOI:** 10.21956/hrbopenres.15633.r53862

**Published:** 2026-06-05

**Authors:** Anneliese Willems

**Affiliations:** 1 University of Melbourne, Melbourne, Australia

Thank you for the opportunity to review this protocol. It is a clear and well-structured submission addressing an important clinical question.

 I have provided my comments below in dot-points to aid with readability: 

**Overall comments:**

- It is a clear protocol addressing an important clinical question
- The rationale and objectives are well articulated, and the use of a scoping review methodology is appropriate given the heterogeneity of the literature.

**Major comments:**

- **Strength of key claim regarding GP capability**
  - The statement: *“Given their expertise in contraceptive management, mental health support and ability to provide continuity of care, GPs are well-positioned to prescribe and monitor isotretinoin”* (ref 17) appears stronger than the supporting evidence
  - The cited study demonstrates GP interest and identifies enablers, but also highlights variability in practice and gaps in monitoring and contraceptive management
  - Consider moderating this statement or supporting it with additional evidence
- **Definition and scope of grey literature**
  - While the protocol states that grey literature will be included, it does not define what will be considered eligible
  - Given the heterogeneity and variable quality of grey literature, this lack of definition may introduce inconsistency and selection bias
  - Clarification is needed regarding:
    - Types of grey literature to be included (e.g. guidelines, policy documents, organisational reports, conference abstracts, theses, tiktoks, podcasts reels, social media etc)
    - Any limits on source type, quality, or jurisdiction
- **Grey literature search strategy**
  - The approach of including the “first 100 results” from Google Scholar and Web of Science is not sufficiently justified
  - Search engine ranking algorithms are not transparent or reproducible (to my understanding), which may introduce bias
  - Consider:
    - Providing a rationale for this cutoff
    - Supplementing with targeted grey literature sources (e.g. guideline repositories, professional bodies)
- **Data extraction methodology**
  - The data extraction process is described at a high level, but could be expanded to improve reproducibility
  - In particular, it would be helpful to specify:
    - Whether the extraction tool will be piloted
    - How consistency between reviewers will be ensured (e.g. double extraction of a subset)
    - How disagreements will be handled beyond discussion
  - Consider whether a systematic review platform (e.g. Covidence or similar) will be used to support data management, noting that this is a scoping review but these platforms may still have a place in streamlining screening. 

**Minor comments / suggestions:**

- **Referencing style**
  - There appears to be consistent over-referencing throughout (e.g. multiple references per sentence where not required) - *this can reduce readability and may not add additional value*
  - Consider streamlining references to key supporting sources
- **Population definition**
  - The protocol focuses on GPs, but it is unclear whether GP registrars/trainees will be included - clarifying inclusion of trainees may be important, particularly as they may represent a distinct group in terms of prescribing practices and confidence
- **Research team description**
  - Affiliations and roles are provided, but the disciplinary backgrounds of the authors are not explicitly described
  - Given the interpretive nature of scoping reviews, outlining the perspectives represented (e.g. general practice, pharmacy) would enhance transparency and help contextualise interpretation
- **Terminology / consistency**
  - The protocol alternates between “GPs”, “general practitioners”, “primary care doctors”, and “family medicine physicians”. This is reasonable, but it may help to define these terms more clearly at the outset and state how international variations in role/title will be handled during screening and synthesis.
- **Potential overlap with existing review**
  - Reference 22 is cited as a scoping review on competencies and clinical guidelines for managing acne with isotretinoin in general practice. It may be worth clarifying more explicitly how the present review differs from that work.
- **Conceptual boundaries**
  - It would be helpful to clarify whether this review is focused only on GP initiation of isotretinoin, or whether it also includes shared care, continuation prescribing, and monitoring undertaken in primary care after non-GP specialist initiation. These are distinct models of care and may affect interpretation of findings.
- **No date restrictions**
  - Inclusion of studies without date restriction is defensible, but older isotretinoin literature may reflect substantially different prescribing rules and monitoring practices. The authors may wish to comment on how temporal changes in practice will be considered during synthesis.

**Optional idea: **

- The authors may wish to briefly outline how they will approach synthesis of heterogeneous evidence, particularly given the inclusion of both peer-reviewed and grey literature
  - For example, whether findings will be grouped by theme (e.g. attitudes, barriers, prescribing practices, system factors)

Is the study design appropriate for the research question?

Partly

Is the rationale for, and objectives of, the study clearly described?

Yes

Are sufficient details of the methods provided to allow replication by others?

Partly

Are the datasets clearly presented in a useable and accessible format?

Not applicable

Reviewer Expertise:

General practice, dermatology, clinical education, and primary care health systems. Experienced in qualitative research and scoping review methodology.

I confirm that I have read this submission and believe that I have an appropriate level of expertise to confirm that it is of an acceptable scientific standard.

# Reviewer report: Reviewer response for version 1

**DOI:** 10.21956/hrbopenres.15633.r54465

**Published:** 2026-04-25

**Authors:** Cathrine Strom Christiansen

**Affiliations:** 1 University of Oslo, Oslo, Norway

**The Management of Acne in General Practice using Isotretinoin: A Scoping Review Protocol**

 Thank you for the opportunity to review this scoping review protocol. Acne is a common topic in general practice, and I appreciate that the authors have chosen to address it. This scoping review is timely in light of increasing antimicrobial resistance (AMR) and therefore the need for non-antibiotic treatment, and familiar inequity in access for isotretinoin for patients.

 Overall, I find the protocol clear, well written, and easy to follow, with good flow. It adheres to the recommendations in the JBI Manual for Evidence Synthesis (2024) and other guidance on scoping reviews as a method.

 It appears that a scoping review is an appropriate choice here. However, I wonder if the authors could make an argument for why a scoping review is preferred over a systematic review. Is the present scoping review intended as a precursor to a later systematic review? Clarifying this rationale would strengthen the methodological justification.

 The tables are well structured and easy to understand.

 In the Introduction, I would suggest adding a reference in the third paragraph to support the statement that patients with acne are primarily managed in primary care.

 In the Inclusion criteria section, the descriptions of “Concept” and “Context” are generally good, but the reader must pay close attention not to interpret the second sentence of each paragraph as essentially saying the same thing. It may be helpful to rephrase these parts to more clearly highlight the differences between “Concept” and “Context.” I would also suggest defining the term “grey literature” the first time it is used, as not all readers will be familiar with this concept.

 In the Methods and search strategy section, the initial search in the three-step search strategy is described as having been conducted in MEDLINE, i.e. a single database. According to the JBI Manual for Evidence Synthesis (2024), the initial search should preferably be conducted in at least two databases. It is possible that the authors plan to include additional databases in the actual scoping review, but this is not clearly stated. I recommend clarifying this point and, if only one database is to be used initially, providing a brief justification.

 In the third paragraph of the Search strategy section, the authors state: “Following the identification of all relevant keywords, search terms, and controlled vocabulary, the search strategy will be finalised (…). This search strategy, and any variations, will be documented and reported in full in the final scoping review.” The fact that the search strategy is not fully finalised before the review starts opens up a potential for selection bias. This may be less problematic in a scoping review than in a systematic review, but it would be helpful if the authors briefly acknowledged and commented on this in the protocol.

 I would prefer that the draft data extraction tool was described in more detail in the protocol. One option would be to develop Appendix B into a table that could also be as a table in the final article, rather than only as an appendix. My impression is that there is substantial additional information in the draft data extraction tool that is not currently reflected in the manuscript. This could be described more fully under the “Data extraction” section, particularly after the phrase “findings relevant to the review questions.”

Is the study design appropriate for the research question?

Yes

Is the rationale for, and objectives of, the study clearly described?

Yes

Are sufficient details of the methods provided to allow replication by others?

Yes

Are the datasets clearly presented in a useable and accessible format?

Not applicable

Reviewer Expertise:

My area of expertise is acne management in general practice and registry-based studies.

I confirm that I have read this submission and believe that I have an appropriate level of expertise to confirm that it is of an acceptable scientific standard, however I have significant reservations, as outlined above.

# Reviewer report: Reviewer response for version 1

**DOI:** 10.21956/hrbopenres.15633.r53861

**Published:** 2026-03-06

**Authors:** Weeratian Tawanwongsri

**Affiliations:** 1 Walailak University, Nakhon Si Thammarat, Thailand

1. Introduction: The content is disjointed, with limited linkage between paragraphs. The narrative also shifts back and forth among prevalence, management, and prognosis. The authors should consider restructuring the section to flow from background information to current knowledge, then to the identified knowledge gap, and finally to the study aim.

 2.1 Methods: Some study designs should be excluded, including case reports, case series, and review articles.

 2.2 There is a mismatch between the stated review focus and the population definition in Table 1. The protocol repeatedly frames the review as mapping GPs’ use of isotretinoin (clinician attitudes/practices), but Table 1 defines the “Population” as patients with acne in primary care settings, which does not align with the intended unit of analysis (GPs/practice-level evidence).

 2.3 In the data extraction section, the authors may revise the extracted items to align with the stated objectives.

 2.4 The search strategy (Table 2) does not include any isotretinoin-related terms.

 2.5 Web of Science should not be described as “grey literature”; it should be listed under bibliographic databases.

Is the study design appropriate for the research question?

Yes

Is the rationale for, and objectives of, the study clearly described?

Yes

Are sufficient details of the methods provided to allow replication by others?

Partly

Are the datasets clearly presented in a useable and accessible format?

Partly

Reviewer Expertise:

Dermatology, Internal Medicine

I confirm that I have read this submission and believe that I have an appropriate level of expertise to confirm that it is of an acceptable scientific standard, however I have significant reservations, as outlined above.
